# Information Entropy Metrics to Address the Complexity of Cooperative Gating of Ion Channels

**DOI:** 10.3390/e28020197

**Published:** 2026-02-10

**Authors:** Agata Wawrzkiewicz-Jałowiecka, Paulina Trybek, Michał Wojcik, Przemysław Borys

**Affiliations:** 1 Department of Physical Chemistry and Technology of Polymers, Silesian University of Technology, Strzody 9, 44-100 Gliwice, Poland; 2 Institute of Physics, University of Silesia in Katowice, 40-007 Katowice, Poland; paulina.trybek@us.edu.pl; 3 Faculty of Biomedical Engineering, Silesian University of Technology, Roosevelta 40, 41-800 Zabrze, Poland

**Keywords:** ion channels, cooperative gating, Shannon entropy, sample entropy, channel cluster, channel cooperativity model

## Abstract

Ion channels in biological membranes can form spatially localized clusters that exhibit cooperative gating behavior. In this mode, the activity of one channel modulates the opening probability of its neighbors. Understanding such inter-channel interactions is key to elucidating the molecular mechanisms underlying electrochemical signaling and advancing channel-targeted pharmacology. In this study, we introduce a simplified stochastic model of multi-channel gating that allows for systematic analysis of cooperative behavior under controlled conditions. Two information-theoretic metrics, i.e., Shannon entropy and Sample Entropy, are applied to simulated multi-channel datasets, including idealized total current traces and dwell-time sequences of cluster states, to quantify inter-channel cooperativity. We show that the entropic measures display a strong dependency on the strength and type of cooperation (non-, positive, or negative cooperation). The proposed entropy-based framework offers a generalizable and quantitative approach for biomedical data analysis, demonstrating effectiveness in interpreting multi-channel recordings and uncovering cooperative mechanisms in ion channel behavior. The underlying mechanisms by which entropy reflects cooperativity are expected to appear in real recordings, where deviations can further aid in characterizing individual channel features in future work.

## 1. Introduction

The activity of ion channels, being membrane proteins responsible for the rapid and selective passage of ions such as Na^+^, K^+^, Ca^2+^, or Cl^−^ down their electrochemical gradients, is crucial for the regulation of cellular ion homeostasis and signaling. For this reason, ion channels constitute a prominent group of molecular drug targets [1,2,3,4,5,6,7,8].

Ion channels do not exhibit a constant permeability to specific ions. Instead, their conductance is governed by conformational dynamics that enable transitions between conducting (open) and non-conducting (closed) states, a process known as channel gating [1,9,10]. The probability and kinetics of these transitions vary and generally depend on multiple factors. Based on the primary stimulus that promotes channel opening, ion channels are commonly classified as voltage-gated, ligand-gated, or mechanically gated [1].

Ion channels constitute a vast and diverse family of proteins, typically named based on their ion selectivity, principal activating stimuli, and basic structural and functional characteristics. The sensitivity of a given channel type to activating or deactivating stimuli can be further modulated by alternative mRNA splicing [11,12,13,14,15,16] and post-translational modifications [17,18,19,20,21,22]. These regulatory mechanisms fine-tune the behavior of a given channel type to its specific biological role within a particular cell, membrane environment, and set of external conditions [23].

Beyond these well-established mechanisms, an important yet still relatively poorly understood phenomenon that may further amplify the functional repertoire of ion channels is cooperative gating [24,25,26]. By modulating channel responsiveness through interactions between neighboring channels, cooperative gating can profoundly influence cellular electrical signaling. This phenomenon constitutes the central focus of the present work.

Some ion channels tend to self-organize in cellular and organelle membranes in the form of clusters composed of channels of the same type [27,28,29,30]. Within such clusters, channels can interact with each other and collectively modulate their gating behavior. Depending on the intrinsic nature of a particular ion channel type, the resulting collective gating can be positive or negative. Positive coupling enhances channel-mediated signaling. In these terms, the opening of one channel from the cluster increases the opening probabilities of all its neighbors forming the same cluster (e.g., [31,32,33]). Channel coupling can also be negative, where the opening of one channel decreases the chances of its neighbors to open, effectively reducing channel-generated ionic currents (e.g., [34,35]). There are multiple examples of cooperative gating in the literature including the K^+^ channels, like S6 KvAP peptide channels [36], KcsA [30,37], K_*ATP*_ [34], Slack [33,38,39], Kv2.1 [35], Na^+^ channels [31], pores formed by Na_*V*_1.5 α-subunits [40], Ca^2+^ channels, e.g., Ca_*V*_1.2 [32,41], Ca_*V*_1.3 [28,42], glycine-gated chloride channels [43], ryanodine receptors 1 (RyR1 channels) [44], voltage-dependent anion channels [45], nicotinic acetylcholine receptors [46], and ionotropic purinergic receptors P2X2 [47]. Across all these channels, the physiological, pathological, and pharmacological consequences of cooperative gating are substantial, including, e.g., disease-related mutations that enhance positive collectivity effects [39]. This underscores the need for further focused investigation of this phenomenon. In this work, we consider the cooperative gating within the clusters of channels of the same type [24], in contrast to some of the related problems like the inherent cooperativity within the intra-channel subunits [48,49,50] or the concerted action of the ion channels of different types [51,52].

Most investigations of collective gating rely heavily on comparing the current characteristics of multi-channel clusters with a hypothetical response obtained by summing independent single-channel signals [10]. Since the opening probabilities are not fixed and depend on channel activation in an unknown way, we cannot easily apply binomial distributions to obtain estimates of even simple quantities as the number of channels in the patch itself. The only reliable estimate (frequently applicable) is the maximum number of open channels found in a sufficiently long record. A comprehensive understanding of channel cooperativity requires a more detailed analysis of multi-channel data than simple changes in the opening probability in channel clusters, encompassing the presence, strength, and temporal range of internal correlations, recurring patterns, characteristic time scales of concerted channel opening and closing, and the overall complexity of the recorded signals. Such information may have a high physiological significance, and it could not only facilitate the diagnosis of cooperative gating (particularly in situations where experimental data are limited) but also provide valuable constraints for the development of physically and biologically plausible models of the molecular mechanisms underlying inter-channel communication.

Existing analytical approaches developed for single-channel data are not readily transferable to multi-channel recordings, primarily because the responses of individual channels within a cluster cannot be measured independently [9,10]. In channel clusters, only the superimposed collective response is experimentally accessible, which poses a substantial challenge to conventional data analysis methods. The superimposed and aggregated form of the data also limits the applicability of existing methods used to analyze interrelated datasets, such as correlation analysis, mutual information, and coherence analysis [53]. These methods could be utilized to describe how a single channel or a multi-channel cluster reacts to the controlled changes in the environment [54,55], but not to directly assess whether and how each channel from a given cluster interacts with its neighbors (since, let us repeat, only a superimposed signal is available). This situation motivates the development of new analytical frameworks or, at least, the enhanced critical assessment of the applicability of existing ones.

In this context, the present work focuses on evaluating the applicability of selected information-theoretic measures for the analysis of time series describing multi-channel activity (in a typical superimposed form). Channel clusters exhibiting different strengths and modes of inter-channel cooperation are expected to produce electrical signals that reflect varying degrees of independent vs. concerted channel action. Thus, the internal complexity and the entropic traits of these signals should differ substantially.

In recent years, information theory has become a pivotal tool for quantifying the complex dynamics of ion channels, utilizing diverse entropic measures to characterize their behavior. To address systems with long-range correlations and physical cooperativity, such as coupled mechanotransduction channels, researchers have successfully applied Tsallis entropy, a non-extensive realization that accounts for subadditive or superadditive behaviors [56]. Furthermore, the rate of information gain, derived from relative Kullback entropy, calculated between the voltage signal and the output current signal serves as a unifying metric for identifying stochastic resonance in single channels, demonstrating how noise can paradoxically enhance signal detection [57]. Beyond these, the entropy production rate is increasingly monitored to describe the thermodynamic landscape of channels operating far from equilibrium, linking microscopic gating events to macroscopic collective phenomena [58]. In this work, we decided to apply two different entropy-related metrics, i.e., Sample Entropy (SampEn) [59,60,61,62] and Shannon entropy [62,63,64], to the simulated multi-channel data to show how sensitive these characteristics are to the symptoms of inter-channel cooperativity.

Intuitively, Shannon entropy describes the diversity of the distribution of a given variable. Thus, the more versatile values it can take with considerable frequency (that is, the more different measurable and significantly occupied states), the higher Shannon entropy [62]. In turn, Sample Entropy is sensitive to the occurrence of repetitive patterns within the analyzed series. The more regular the signal, i.e., the more there are similar patterns of data points that remain similar when one more point is added to the length of the considered pattern, the lower the SampEn value [61,62]. From this perspective, Shannon entropy and Sample Entropy, being two complementary information-theoretic measures, emerge as promising diagnostic tools for identifying and classifying cooperative ion channel systems.

In fact, these two entropic metrics have already been successfully used in ion channel research to describe channel gating [62,65,66,67,68]. In the work of Machura et al. [65], the authors applied Sample Entropy and its extension in the form of a Multiscale Entropy (MSE) to the single-channel patch-clamp recordings of mitoBK (mitochondrial BK channel) from human dermal fibroblasts to classify the channel behavior according to the membrane potential at which the signals have been obtained. In addition, they considered how the values of entropy change with the signal’s sampling frequency [65]. SampEn and MSE values can be used to distinguish between the activity of different mitoBK channel variants present in different cell types [68]. In [62], Shannon and Sample Entropy have been applied to the experimental series that describes the single-channel activity of the large-conductance, voltage- and Ca^2+^-activated potassium channels from the plasma membrane of human bronchial epithelial cells at different concentrations of Ca^2+^ and/or quercetin. The values of these entropies were helpful in formulating hypotheses about the interdependencies of channel activation by both agents, including their synergy/competition and plausible hydrophobic gating scenarios. In that work, further applications of entropic analysis in other channel-oriented problems, such as channel activation by some small ligands or ball-and-chain inactivation, have also been discussed. Another work of this group [66] considers the simple two-state model of ion channel gating based on the overdamped Langevin dynamics. Shannon, Spectral, Sample, and Slope entropies were evaluated for the results of model simulation (corresponding to the time series of single-channel ionic currents) at different values of temperature and steepness of the potential wells corresponding to the open and closed channel states. The results describe the sensitivity of the mentioned entropic metrics representing different aspects of gating complexity to the variation in energy landscapes and noise. In the work of Lisowski et al. [58], the concept of Shannon entropy has been creatively extended to the stochastic thermodynamic description of entropy production in a single-channel model system (affected by abrupt perturbations of the system by the environment and exhibiting a number of possible transient ”semi” states and stable states) and its extension to a group of synchronized cooperative channels. Finally, the recent article of Akilli et al. [67] shows the application of Sample Entropy to the whole-cell recordings of two different types of ion channels, EAG1 in MCF-7 cells and TRP channels in ARPE-19 cells at different membrane potentials. The author assessed the ion channels’ functionality according to the entropy, and they observed that the maximal entropy has been reached near equilibrium, where the ionic fluxes have minimal amplitudes. In summary, according to the aforementioned literature [58,62,65,66,67,68], the information entropy and the related metrics are versatile analytic tools for investigating channel-based systems. In the current work, we present for the first time how Shannon and Sample Entropy can be applied to the data that describe the activity of a multi-channel cluster, and we show the main possible interpretation pathways.

For this purpose, we employ a simple toy model of single- and multi-channel activity to generate predictions for information-theoretic entropy measures applied to current recordings of ion channel clusters under controlled channel cooperation. The analysis is restricted to the information entropy estimates derived from current time series, rather than thermodynamic entropy of the channel system [69]. Entropy is also evaluated for the associated dwell-time series corresponding to resolved states of individual channels and multi-channel clusters.

We expect the SampEn and Shannon entropy to highly depend on cluster size and the corresponding change in the strength of inter-channel cooperation. In particular, according to the existing literature, Shannon entropy characterizes the current histogram particularly well (where the number of channels manifests itself in the number of maxima). On the other hand, SampEn applied to the dwell-time series measures transitions between states, and the frequency of these transitions depends on the number of active channels in the cluster. Here, we decide to calculate Shannon entropy for the two considered types of data (current series and dwell-time series) and SampEn for the dwell-time series, and we comment on the sensitivity of the calculated entropies to different inherent features of the analyzed data, which can guide future channel-oriented research.

Our purely theoretical approach enables analysis in a fully controlled setting, where we can tune the size of the cluster, strength of the channel-activating stimuli, and the mode and strength of inter-channel cooperation. Such an approach allows us to focus on idealized regimes of channel cluster operation. Thereby, it facilitates the identification of general trends and the straightforward interpretation of results. In practice, however, such well-defined regimes are not expected experimentally due to biological variability. However, being aware of them allows for focusing the analysis on the channel-specific features of the recorded signals. By working with simulated data, we systematically examine how controlled changes in the gating dynamics of a channel cluster, particularly the mode and strength of inter-channel cooperation, including cluster size-related effects, influence Shannon and Sample Entropy values. Thus, we aim to develop an intuition that can be directly applied to the analysis of real multi-channel patch-clamp recordings [10], where we will be able to dissect the general trends from channel-specific signatures, which are of the greatest interest.

## 2. Materials and Methods

### 2.1. Simulation of Ion Channel Cluster Activity

In this work, we simulated the activity of a channel cluster comprising $N_{ch}$ channels of the same type, with $N_{ch}$ ranging from 1 to 6. In that aim, we revisited and appropriately re-formulated the random walk-based model proposed in [70]. In this approach, for each channel within the cluster, its gating is described by a discrete random walk process performed by the reaction coordinate (RC) over the one-dimensional conformational space.

The concept of the reduction of the complex structure of the channel gate into the 1D movement of an abstract variable has been known in the literature for some time [71], and reflects the transition sequence of conformational states that the ion channel has to pass to switch from closed to open and vice versa. A simple measure of the activation level of the ion channel, which can work as a reaction coordinate, is the opening probability.

**Figure 1:**
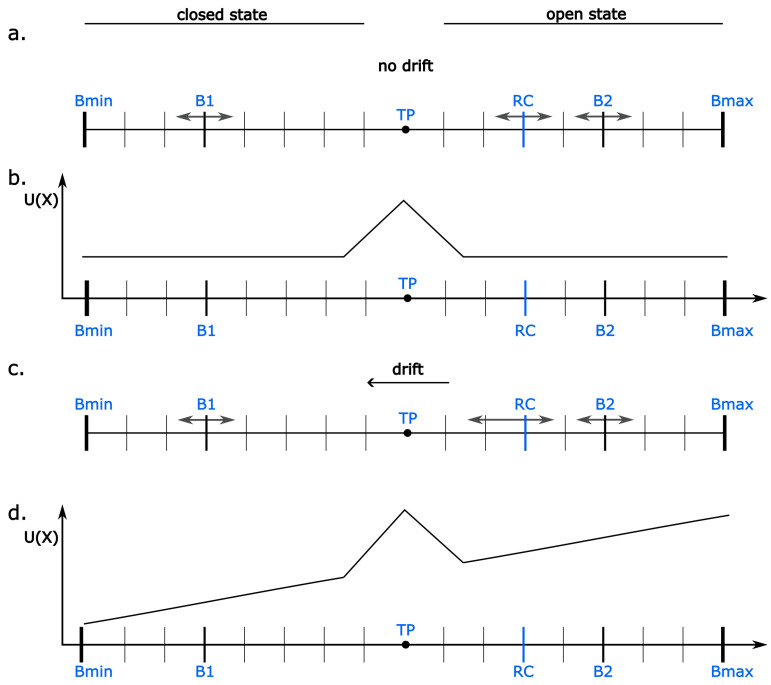
A schematic representation of the model system. (**a**) Each channel is represented by a reaction coordinate (RC). The RC position within its diffusive space is restricted by two moving boundaries B1 and B2 and corresponds to either an open (conducting) or a closed (non-conducting) macro-state of the channel. At half-activation, the open and closed states are equiprobable (there is no global drift toward open/closed states in the model system). (**b**) The schematic representation of the shape of the potential function (*U*). At half-activation, it is flat beside the direct vicinity of the threshold point (TP), which separates open and closed states. In a non-cooperativity mode, in these terms, the probabilities of RC to jump to the left or to the right are equal. (**c**) When there is a non-zero drift, acting in the direction down the potential energy gradient, RC has a greater probability to jump in the direction of the drift. This represents the tendency of a channel to occupy either open or closed states depending on its activation level by some channel-modulating factors (activators/inhibitors). (**d**) The representation of the potential function in terms of a non-zero drift.

The movement of the RC in our model is restricted by two boundaries located symmetrically in relation to the threshold point (TP) separating open and closed states (Figure 1). These boundaries (B1 and B2) may represent the impact of the membrane surroundings on the channel protein functioning. Their variations in time, which extend or reduce the conformational space available for the RC diffusion, allow the model to reproduce the experimentally confirmed Hurst memory effect [70]. The moving boundaries represent the thermal fluctuations of the membrane and the occurrence of transient internal strains within it. The B1 and B2 motions proceed at a larger time scale than the motion of the RC ($D_{RC}/D_{B}$ determined by the ratio of the diffusion coefficients of reaction coordinate and boundaries), which is justified by the difference in mass between the pore-gate domain and its membrane surroundings. The movements of B1 and B2 to extend or shrink the diffusive space of RC are assumed to be equiprobable, and their maximal amplitude is given as $\left|B1\right|=\left|B2\right|=2B_{max}$.

The function of the RC potential (*U*) shapes the probability of the RC to jump to the left (*q*) or to the right (*p*) on the 1D lattice. It is given according to the formula [72]:

(1) $$q=\frac{1}{2}+\frac{\Delta U}{4kT}$$

and

(2) $$p=\frac{1}{2}-\frac{\Delta U}{4kT}$$

where *k* is the Boltzmann constant, *T* is the absolute temperature, and ΔU is a potential energy difference within a lattice step centered around the RC.

The *U* function has a peak at the TP, which illustrates an energetic barrier separating open and closed conformations. This peak is determined by the *B* parameter of the model (Equation (3)) and effectively represents a barrier of large-scale structural reorganization needed to effectively open or close the pore to the K^+^ transport.

Different channel-activating effects, that in the real system are exerted by e.g., voltage and ligand sensors, are represented in the model by a drift force (*A*) contributing to the energetic landscape of the available RC positions. In the simplest considered case, the drift force is constant (i.e., the sensors induce a ramp potential contribution), and its amplitude depends on the sensors’ activation level. Such a representation aligns the allosteric picture of the system, in which sensor activation exerts an open-enhancing effect, but it is not mandatory for pore opening.

Since we adapt the original model proposed in [70] to enable the description of a cluster of ion channels of the same type, we introduce additional assumptions. In each step of the simulation, each channel from a cluster performs its random walk on its own lattice. The instantaneous size of the lattice is the same for all $N_{ch}$ channels, and is limited by the same (global) boundaries B1 and B2. Since we wish to model different modes and strengths of inter-channel interactions, we propose that the motion of each $RC_{i}$, representing one channel of a considered cluster, is affected by the functional states of other *RC*s (i.e., by all $RC_{j}$, where $j\in [1,\ldots ,N_{ch}]$ and j≠i, representing the neighboring channels). We incorporate this idea into the model by introducing an appropriate additive component of the potential function gradient, i.e., $N_{OpenNeighbors}\cdot \Delta_{drift}$. In this way, the opening of one channel affects the potential of all other channels co-assembled in a given cluster. If $\Delta_{drift}$ is negative, open states are facilitated, and the opening of one channel supports the opening of its partners (”positive cooperation”). If $\Delta_{drift}$ is positive, closed states are facilitated after any channel in the cluster opens (”negative cooperation”). If $\Delta_{drift}$ equals zero, the channels gate independently (”no cooperation”). Alternative formulation could involve the modulation of boundaries B1 and B2 in response to cooperation, which is a possible interaction mechanism, but not the only one. The inter-channel cooperation within a cluster may involve multiple mechanistic components, each with varying degrees of specificity to a particular channel–membrane system. Collective gating effects can arise from diverse mechanisms, including chemical signaling molecules, electrostatic or hydrophobic interactions, and long-range allosteric couplings [24]. Given this mechanistic diversity and the potential additive contributions of physical processes acting across different spatial and temporal scales of inter- and intra-channel activation, complex nonlinear behaviors may emerge within the overall framework of cooperative gating.

In this work, for clarity and generality, we model the inter-channel cooperation in the simplest possible way, which frequently can serve as an approximation to more complex processes—as an additive drift component linearly dependent on the number of transiently open channels within the cluster. The precise nature of this external force is intentionally left unspecified, allowing flexibility for interpretation and adaptation of the model to specific channel systems in future studies.

In each step of the simulation, for each channel in a cluster, its potential function $U_{i}$ is calculated and takes the form:

(3) $$U_{i}\left(x\right)=\left\{\begin{array}{c} \left(x-B1\right)\cdot \left(A+N_{OpenNeighbors}\cdot \Delta_{drift}\right)+U_{B1};x\in \left[B1;TP-1.5\right)\\ \left(x-\left(TP-1.5\right)\right)\cdot B+U_{TP-1.5};x\in \left[TP-1.5;TP\right)\\ \left(x-TP\right)\cdot \left(-B\right)+U_{TP};x\in \left[TP;TP+1.5\right]\\ \left(x-\left(TP+1.5\right)\right)\cdot \left(A+N_{OpenNeighbors}\cdot \Delta_{drift}\right)+U_{TP-1.5};x\in \left(TP+1.5;B2\right] \end{array}\right.$$

where B1 and B2 are the locations of the left-hand and right-hand boundaries, TP is the threshold point position, $U_{B1}$, $U_{TP}$ $U_{TP-1.5}$ denote the potential values at given points TP and TP−1.5, and *A* represents the drift force toward the open or closed states and is a potential slope between points B1 and TP−1.5 (and between points TP+1.5 and B2).

(4) $$A=\frac{U_{TP-1.5}-U_{B1}}{TP-1.5-B1},$$

*B* represents the potential barrier between the open and closed states and is defined by a potential slope between points TP−1.5 and TP.

(5) $$B=\frac{U_{TP}-U_{TP-1.5}}{TP-(TP-1.5)}.$$

In the first step of the simulation, we randomly set the positions of the *RC*s of all the channels that form a cluster. From this moment on, the corresponding potentials are calculated at each step of the simulation, and based on their values, each *RC* jumps to the left or to the right with probabilities given by Equations (1) and (2) (but it can never jump over the boundaries B1 and B2; the TP point also cannot be reached by RC—it can just jump from TP−1 to TP+1 or reverse). The time between two subsequent steps of the simulation is called the ‘reaction coordinate time unit,’ and the distance between consecutive nodes of the lattice (diffusive space of the *RC*s) is called the ‘reaction coordinate unit’ or just ‘rcu’.

The simulation procedure can be summarized as follows:

1. The symmetric lattice with the *TP* point in the middle and maximum number of nodes equal to 4$B_{max}$ on either side is set.
2. The initial positions of the boundaries B1 and B2 are set to $-B_{max}$ and $B_{max}$.
3. The initial positions of all $N_{ch}$ reaction coordinates *RC*s are randomly chosen between B1 and B2 (the TP point is excluded).
4. The potential function $U_{i}$ is calculated according to Equation (3) for each RC.
5. The position of each reaction coordinate is randomly changed by one rcu, with the probabilities of movement to the right and to the left given by Equations (1) and (2). (If RC reaches the B1 or B2 positions, it stays in its previous position. If RC=TP−1 and it should move to TP, it jumps to the TP+1. If RC=TP+1 and it should move to TP, it jumps to the TP−1.)
6. The position of each reaction coordinate in relation to TP is checked. If RC is at the right-hand side of the TP, the open state is recognized. Otherwise, the closed state is assigned.
7. The idealized current through a cluster (*I*) is evaluated as the number of open channels in the considered cluster (number of *RC*s > TP). If all channels are closed (all *RC*s < TP), *I* = 0.
8. Steps 4–7 are repeated for a number of time steps determined by the value of $D_{RC}/D_{B}$.
9. The boundaries B1 and B2 are randomly and synchronously moved for one step length toward or away from TP with equal probability. (If B1 reaches −2$B_{max}$ or TP−1 positions, it is reflected to its previous position. Analogously, if B2 reaches 2$B_{max}$ or TP+1 positions, it is reflected to its previous position.)
10. Steps 4–8 are repeated for a desired time series length.

**Figure 2:**
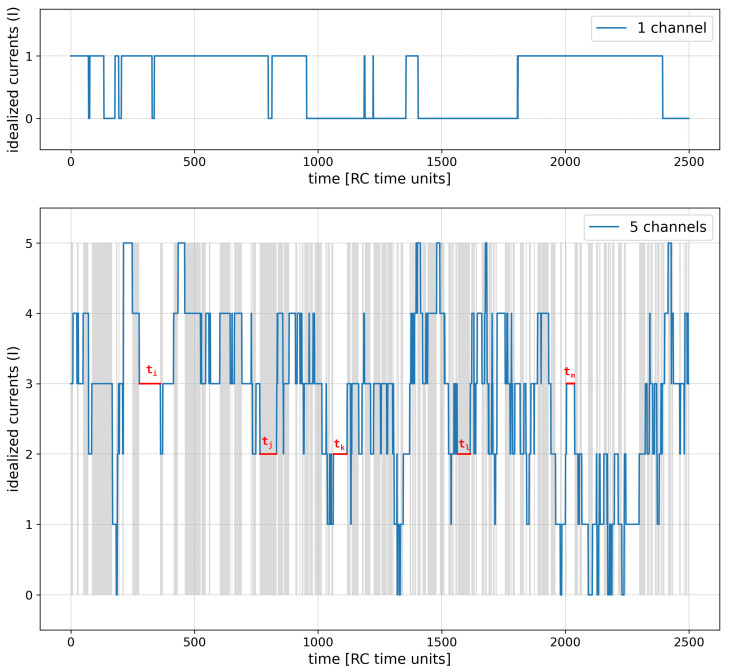
A sample of the idealized currents generated by the model simulation. **Upper panel**: A single-channel variant at half-activation (drift = 0); **lower panel**: 5-channel variant (drift = 0, no cooperation). Example dwell-time periods $t_{i}$, $t_{j}$, $t_{k}$, $t_{l}$, $t_{m}$ are shown; the remaining periods are just delimited by gray/white background stripes for clarity.

The results of model simulation have the form of a time series of idealized currents (*I*) through a cluster (Figure 2). It is called ’idealized’, since the conductivity of each channel from the cluster is considered in a dichotomized (binarized) way—1 represents its open state, 0 represents its closed state. Consequently, for each simulation step, one obtains an integer value ranging from 0 to $N_{ch}$, which directly indicates how many channels were open at this simulation step (Figure 2, lower panel).

The simulated idealized current signals are in fact a superposition of the $N_{ch}$ individual traces of the binarized single-channel activity. Such superimposed series are achievable experimentally using the patch-clamp method when a given patch includes several individual “single” proteins and the corresponding raw recordings of their noisy currents get “idealized” by some available methods [73,74,75]. Our choice of taking the idealized and not the raw multi-channel data was dictated by the possibility of reaching the greatest generality of our analysis aimed to track the changes in entropy as a result of different modes and strengths of inter-channel cooperation, which should not be distorted by the additional effects like different signal-to-noise ratios corresponding to the signals describing various ion channel types.

Based on the obtained *I* series, one can easily calculate the mean value of relative cluster currents $I/I_{max}$ (sum of all simulated channel currents divided by the sum of maximal possible currents for the same series length and cluster size—$N_{ch}$·$N_{simulation-steps}$—which corresponds to an average open state probability for each channel from the cluster. Another commonly used parameter in the description of the multi-channel series is NPo, which is the mean number of channels open throughout a simulation.

In this study, the model parameters were set as $U_{TP}=2.0\;\mathrm{kT}$, $B_{\max}=14$, and the ratio of random walk time scales was $D_{RC}/D_{B}=600$. Simulations were run until a dwell-time series of length N=25,000 was obtained. To explore different activation levels of channels within a cluster—mimicking the effects of external factors such as voltage or ligand concentration—the amplitude of the drift force was varied from −0.7 to 0.7 kT/rcu in steps of 0.1 kT/rcu. Half-activation of the simulated channels occurs at a drift amplitude of 0 kT/rcu.

Based on the simulated ion channel current series, the corresponding dwell times for channel or cluster states were generated. For single-channel states, the dwell-time series represents the durations of consecutive open and closed states. For multi-channel states, it represents the durations over which successive cluster current amplitudes remain constant.

Having obtained total current- and dwell-time series at different $N_{ch}$ and drift parameters for varying cooperation modes according to the $\Delta_{drift}$ value, first we evaluated the metrics describing the effectiveness of the model to mimic different types of cluster cooperation—$I/I_{max}$ (mean value of relative cluster currents) and NPo (mean number of channels being open throughout a simulation). We presented these results in a form of activation curves $I/I_{max}$ vs. drift for a selected value of $N_{ch}$, and then in a form of errorbar plots of $I/I_{max}$ vs. $N_{ch}$ and NPo vs. $N_{ch}$, where we juxtapose the data obtained at different strengths and modes of the inter-channel cooperation. After that, we calculate the Shannon entropy for the total current- and dwell-time series and Sample Entropy for the dwell-time series only. These results are shown in appropriate plots where the entropy is presented either as a function of $N_{ch}$ (at drift fixed on 0) or as a function of drift for $N_{ch}$ = 4.

### 2.2. Sample Entropy Determination

Sample Entropy [59] is a powerful tool to quantize the regularity and complexity of time series. In contrast to Shannon entropy, which is addressed to all symbols occurring in the time record, Sample Entropy allows for investigating and quantizing the behavior of correlated sequences within the record.

The basic algorithm to calculate SampEn can be reviewed as follows:

- Consider a time series $X={\left\{X_{n}\right\}}_{n=1}^{N}$ of *N* data points, and for this time series, construct a set of subsequences of length m<N, $u_{m}\left(i\right)=\left\{x_{i},x_{i+1},\ldots ,x_{i+m+1}\right\}$ for i=1,…,N−m+1.
- To investigate correlations, consider a similarity measure between the sequences. For this purpose, we make use of the Chebyshev distance:
  (6) $$d\left[u_{m}\left(i\right),u_{m}\left(j\right)\right]=\max_{k=0,\ldots ,m-1}\left(\left|x_{i+k}-x_{j+k}\right|\right)$$
  Having two sequences apart from each other by less than *r*, i.e., $d[u_{m}\left(i\right),u_{m}\left(j\right)]<r$, with *r* being the similarity threshold (20% of the dwell-time series standard deviation), we consider them matching (they are satisfactorily similar to each other).
- Using the similarity measure, evaluate the probability of finding matching sequences within the considered time series to a given template sequence $u_{m}\left(i\right)$:
  (7) $$C_{i}^{m}\left(r\right)=\frac{n_{i}^{m}\left(r\right)}{N-m}$$
  where $n_{i}^{m}\left(r\right)$ is the number of sequences that meet the similarity criterion $d[u_{m}\left(i\right),u_{m}\left(j\right)]<q$, and N−m+1 is the number of different sequences of length *m* within the record of length *N*.
- The probability (7) can be averaged over all sequences in the record to give:
  (8) $$C^{m}\left(r\right)=\frac{1}{N-m+1}\sum_{i=1}^{N-m+1}C_{i}^{m}\left(r\right)$$
- The Sample Entropy is defined as:
  (9) $$SampEn\left(m,r,N\right)=-\log\left[\frac{C^{m+1}\left(r\right)}{C^{m}\left(r\right)}\right]$$
  SampEn is a the negative natural logarithm of the conditional probability that two sequences similar for *m* points remain similar for m+1 points. Thus, it is a measure of the loss of correlation. In highly correlated process, this is close to 0, while for a sudden correlation drop, one obtains a positive value.

### 2.3. Shannon Entropy Determination

Shannon entropy is a fundamental quantity in information theory [63,64]. For a sequence of symbols $X={\left\{X_{n}\right\}}_{n=1}^{N}$, one can isolate the values which occur in that sequence as $\hat{X}=\left\{{\hat{X}}_{1},{\hat{X}}_{2},\ldots ,{\hat{X}}_{m}\right\}$, m≤N. The values can be assigned probabilities $P({\hat{X}}_{i})$, which in our case, are measured as the frequency to be found in sequence *X*.

Given the above, we define Shannon entropy as:

(10) $$H=-\sum_{i=1}^{m}P\left({\hat{X}}_{i}\right)\log_{2}P\left({\hat{X}}_{i}\right)$$

Using $\log_{2}$ in this expression allows entropy to be measured in bits. In this study, Shannon entropy is estimated from the histogram of dwell times of cluster states (residence times in states with distinct current levels) or of the idealized total current amplitudes. After rescaling, these histograms yield the probabilities used in (10). For the dwell-time distributions, 100 logarithmically spaced bins were used, covering the range from the global minimum to the maximum dwell-time across all analyzed series. For the current series, seven bins at 0, 1, 2, …, 6 were used, reflecting clusters of up to six channels (0 corresponds to no open channels, and 6 to all channels open).

## 3. Results

### 3.1. Simulation of the Collective Gating Model

**Figure 3:**
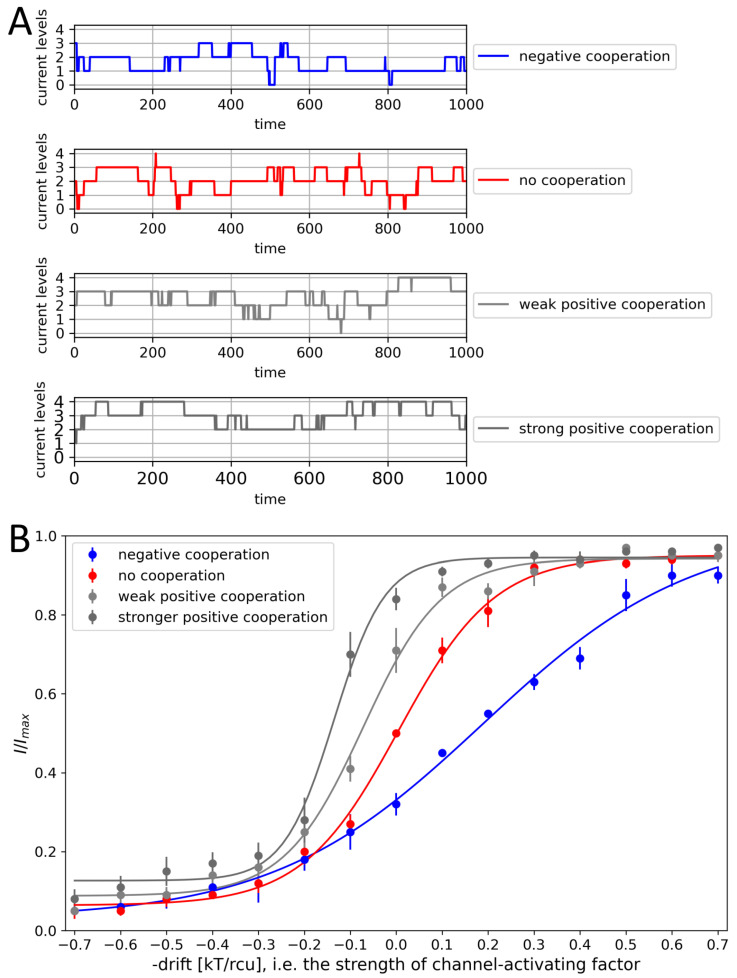
(**A**) Sample excerpts from the total current series obtained for 4-channel clusters of different cooperativities at drift = 0. (**B**) The activation curves obtained as a result of model simulation for 4-channel clusters at different strengths of their mutual cooperation. The results represented by the mean values of relative cluster currents $I/I_{max}$. Red—no cooperation ($\Delta_{drift}$ = 0), blue—negative cooperation ($\Delta_{drift}$ = 0.1 kT/rcu; open state of a channel favors the closure of its neighbors), grey—weak positive cooperation ($\Delta_{drift}$ = −0.05 kT/rcu; open state of a channel favors the opening of its neighbors), dark grey—strong positive cooperation ($\Delta_{drift}$ = −0.1 kT/rcu).

The proposed model is capable of mimicking the results of cooperative behaviors, since the strength and type of coupling modify the signal characteristics (Figure 3A) and the steepness of a channel activation curve (Figure 3B). The positive cooperation shifts the activation curve to the regime typical for lower activation of the channel sensors of external channel-activating factors (to the left in Figure 3). In turn, the negative cooperation leads to the dampening of channel activation by the external stimuli. Thus, the inter-channel enhancement/deprivation may significantly alter the channel-mediated cellular firing properties.

**Figure 4:**
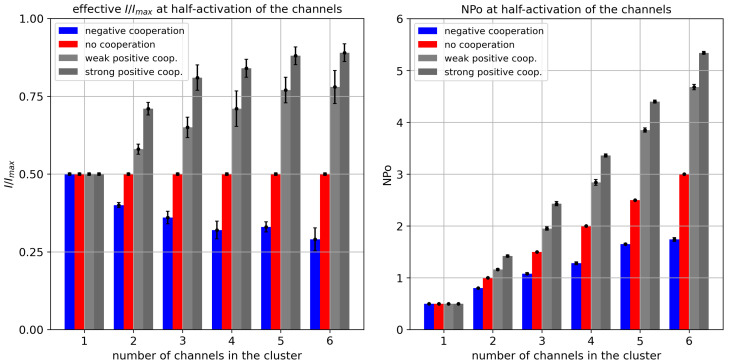
The relative cluster currents $I/I_{max}$ (**left panel**) and mean number of channels open within the analyzed length of simulation, NPo, (**right panel**) for different cluster sizes and different strengths of the inter-channel cooperation. The values of $I/I_{max}$ and NPo were calculated at the half-activation of each channel forming a cluster (drift = *A* = 0). Red—no cooperation ($\Delta_{drift}$ = 0), blue—negative cooperation ($\Delta_{drift}$ = 0.1 kT/rcu; open state of a channel favors the closure of its neighbors), grey—weak positive cooperation ($\Delta_{drift}$ = −0.05 kT/rcu; open state of a channel favors the opening of its neighbors), dark grey—strong positive cooperation ($\Delta_{drift}$ = −0.1 kT/rcu).

To analyze the impact of inter-channel cooperation on crucial metrics describing cluster gating in more detail, we simulated the activity of a single channel and a channel cluster formed by 2–6 channels at half-activation (i.e., for the drift force parameter drift = 0), and then evaluated the corresponding normalized current $I/I_{max}$ and the mean number of open channels NPo (Figure 4). In the positive cooperation mode, the opening of ion channels strongly enhances the probability of all their neighbors to open. In these terms, gating dynamics synchronize to some extent, leading to a more frequent occurrence of the open state of each channel within the cluster. This kind of behavior is straightforwardly dependent on the cluster size because of the assumed additivity of cooperative effects. Consequently, in the positive cooperation mode, the more channels in the cluster, the stronger the open-reinforcing impact, up to a saturation level (where the open state probability exceeds 90%). Analogously, in the negative cooperation mode, where opening of any channel reduces the likelihood of its neighbors to open, the more channels in the cluster, the better pronounced the hampering effects for the ionic transport through the channel cluster.

### 3.2. Shannon Entropy

To rigorously quantify the complexity and the degree of synchronization within these channels, Shannon entropy provides essential information. By characterizing the probability distribution of all possible states within the cluster, this metric serves as a direct measure of the system’s uncertainty and configurational variety. Shannon entropy considers every possible combination of open and closed states of the $N_{ch}$ channels co-assembled within the cluster, capturing how the system distributes itself among various combinations of open and closed states. It acts as a sensitive indicator of the information about the gating process, allowing us to determine which cluster’s behavior is purely stochastic and which is more structured in collective dynamics.

#### 3.2.1. Shannon Entropy of Cluster Currents

**Figure 5:**
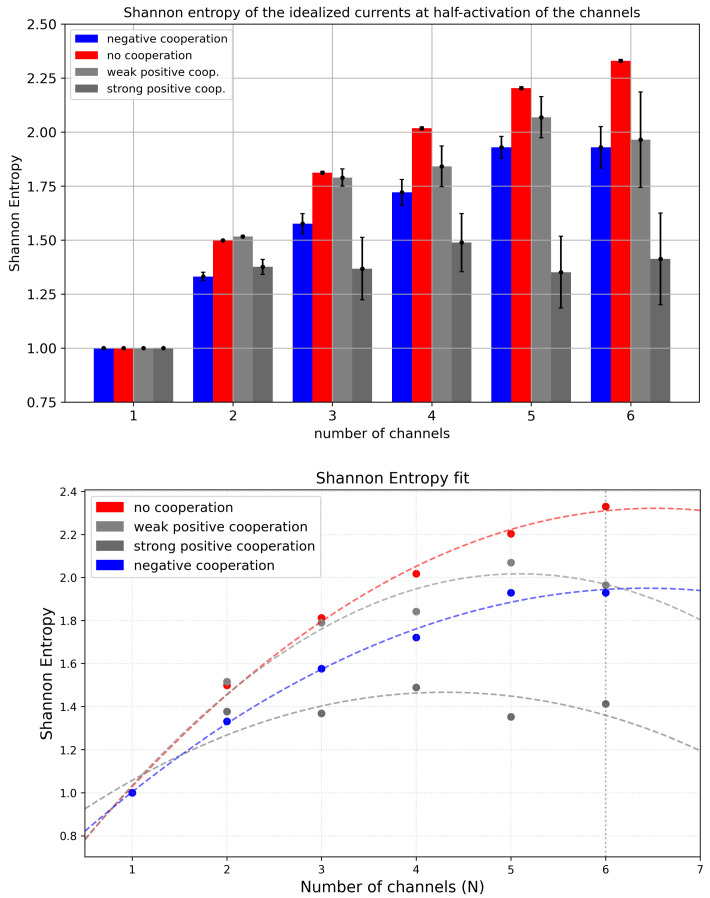
**Upper panel**: Shannon entropy of cluster currents evaluated for different cluster sizes and different strengths of the inter-channel cooperation. The data obtained at the half-activation of the channels (i.e., drift = A = 0). Red—no cooperation ($\Delta_{drift}$ = 0), blue—negative cooperation ($\Delta_{drift}$ = 0.1 kT/rcu; open state of a channel favors the closure of its neighbors), grey—weak positive cooperation ($\Delta_{drift}$ = −0.05 kT/rcu; open state of a channel favors the opening of its neighbors), dark grey—strong positive cooperation ($\Delta_{drift}$ = −0.1 kT/rcu). **Lower panel**: The parabolic fittings of the recognized dependencies of Shannon entropy of cluster currents vs. the number of co-assembled channels for different modes of cooperation. The coefficients of fit are presented in Appendix A.

Shannon entropy measurements for cluster currents (Figure 5, top panel) display expected characteristics: the increase in the number of channels within a cluster introduces new available current levels and enlarges the entropy. However, the trend is not linear and depends on the modality and strength of inter-channel cooperation. In terms of non-cooperative gating within the cluster, one observes a monotonic increase in Shannon entropy with the number of channels that form a cluster. In turn, when the channels cooperate, some of the conduction states become unlikely, and the Shannon entropy increases with $N_{ch}$ until it reaches a saturation level for high $N_{ch}$. Stronger channel cooperation leads to faster stabilization of the Shannon entropy. These dependencies can be well described by parabolic relations (Figure 5, bottom panel). Differences in the evolution of Shannon entropy of the idealized cluster currents with cluster size $N_{ch}$ across datasets with varying inter-channel cooperativity are reflected in the corresponding parabola fitting parameters (Table A1).

**Figure 6:**
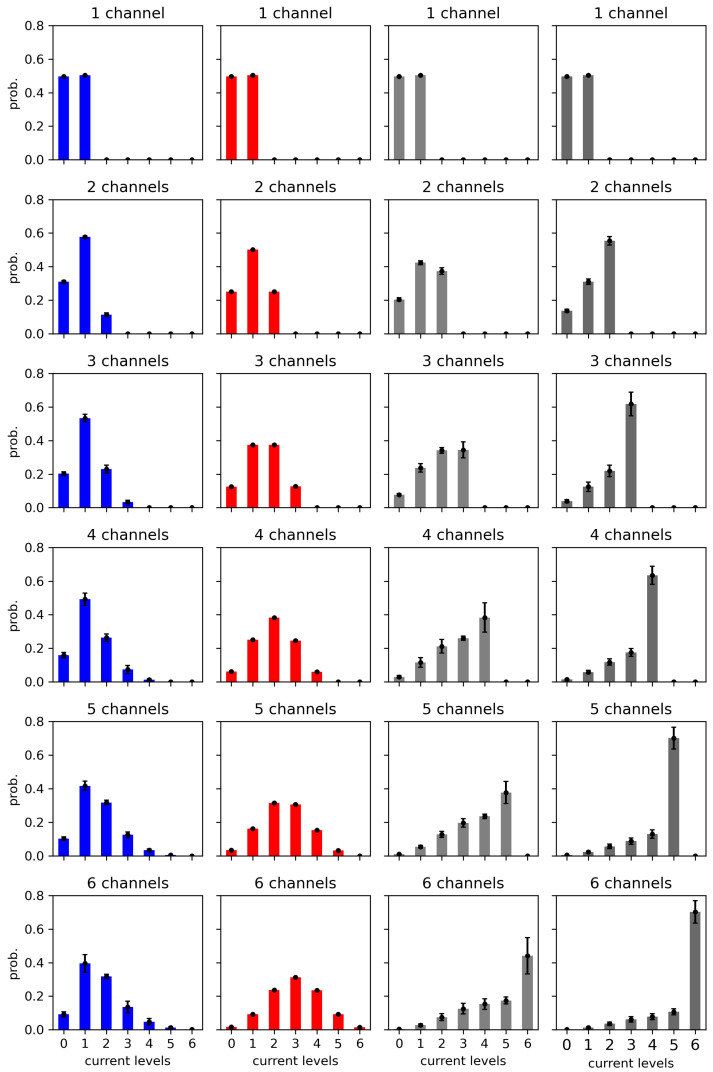
Histograms of cluster currents evaluated for different cluster sizes and different strengths of the inter-channel cooperation. The data obtained at the half-activation of the channels (i.e., drift = A = 0). Red—no cooperation ($\Delta_{drift}$ = 0), blue—negative cooperation ($\Delta_{drift}$ = 0.1 kT/rcu; open state of a channel favors the closure of its neighbors), grey—weak positive cooperation ($\Delta_{drift}$ = −0.05 kT/rcu; open state of a channel favors the opening of its neighbors), dark grey—strong positive cooperation ($\Delta_{drift}$ = −0.1 kT/rcu). Prob. means probability.

**Figure 7:**
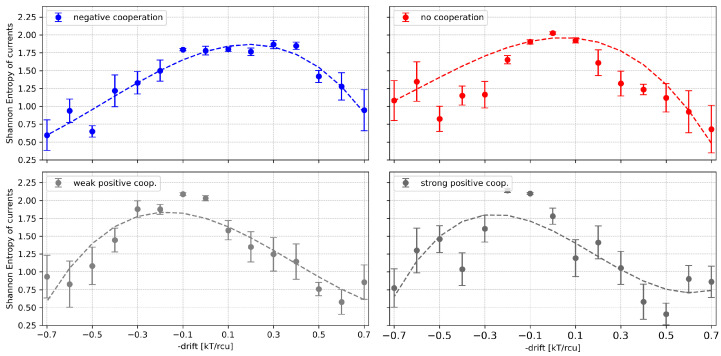
Shannon entropy of time series of total currents flowing through a 4-channel cluster at different values of the global drift (representing the channel activation level by external stimuli, like, e.g., voltage, ligand concentration) and different strengths of the inter-channel cooperation. Red—no cooperation ($\Delta_{drift}$ = 0), blue—negative cooperation ($\Delta_{drift}$ = 0.1 kT/rcu; open state of a channel favors the closure of its neighbors), grey—weak positive cooperation ($\Delta_{drift}$ = −0.05 kT/rcu; open state of a channel favors the opening of its neighbors), dark grey—strong positive cooperation ($\Delta_{drift}$ = −0.1 kT/rcu). For easier recognition of general trends, the results are presented together with their 3-degree polynomial fitting.

The reduction in the probabilities of deeply closed (in the case of positive cooperation) or deeply open (in the case of negative cooperation) current levels is depicted in Figure 6). This effect of cooperation is also seen in Figure 7, where the entropy maximum (corresponding to the maximal complexity reached near the effective half-activation due to drift and cooperation) moves to the left or to the right, depending on the type of cooperation (Table A1).

What is important to note is that the ‘asymmetry’ of how the modality of the cooperation is introduced into the model (the $\Delta_{drift}$-related components depend only on the transient number of open channels, and not the closed ones) has its resemblance in the Shannon entropy outcomes. Introduction of $\Delta_{drift}$ = 0.1 kT/rcu (negative cooperation) exerts lower effects on the Shannon entropy of cluster currents for fixed cluster sizes than the introduction of $\Delta_{drift}$ = −0.1 kT/rcu (positive cooperation) to the model Figure 5.

#### 3.2.2. Shannon Entropy of Dwell Times of Cluster States

**Figure 8:**
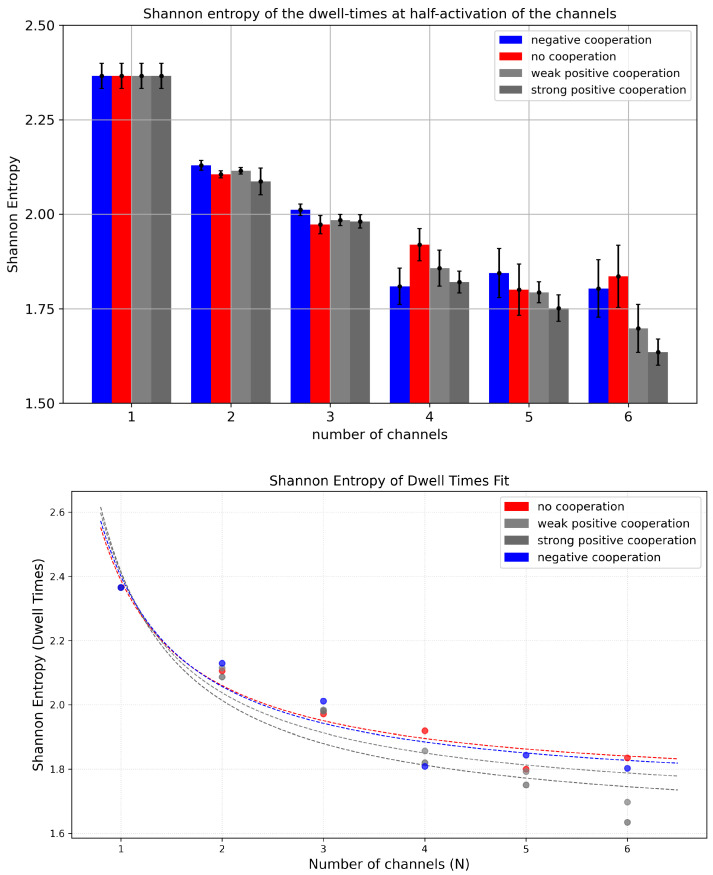
**Upper panel**: The Shannon entropy of dwell times of cluster states evaluated for different cluster sizes and different strengths of the inter-channel cooperation. The data obtained at the half-activation of the channels (i.e., drift = A = 0). Red—no cooperation ($\Delta_{drift}$ = 0), blue—negative cooperation ($\Delta_{drift}$ = 0.1 kT/rcu; open state of a channel favors the closure of its neighbors), grey—weak positive cooperation ($\Delta_{drift}$ = −0.05 kT/rcu; open state of a channel favors the opening of its neighbors), dark grey—strong positive cooperation ($\Delta_{drift}$ = −0.1 kT/rcu). **Lower panel**: The fittings of the recognized dependencies of Shannon entropy of dwell times vs. the number of co-assembled channels for different modes of cooperation. The coefficients of fit are presented in Appendix A.

The behavior of Shannon entropy for dwell-time distributions exhibits a trend opposite to that observed for current distributions (Figure 8). This is because the increase in the number of channels in the cluster allows for an easier escape from a particular conduction state (there are N+1 such states for a cluster with *N* channels). For example, if all *N* channels are open, leaving this conduction state is possible already when only one of them closes. The probability *P* of such an event is *N* times higher than in the case of a single channel dwell-time. All this translates to a dwell-time histogram, which is much more concentrated at short dwell-time values, which reduces the entropy.

**Figure 9:**
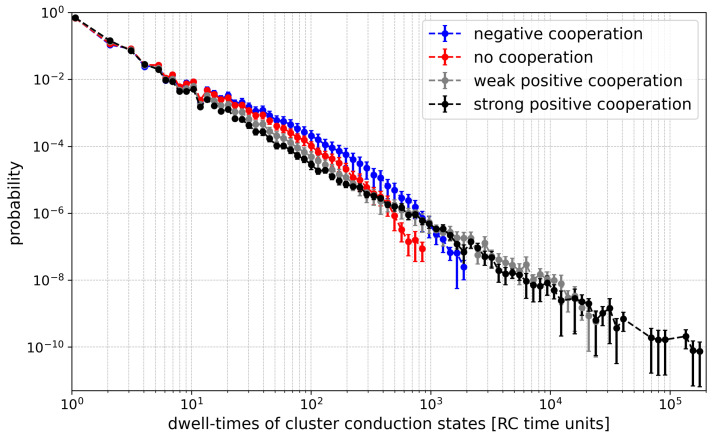
Histograms of dwell-times of cluster states evaluated for different strengths of the inter-channel cooperation at $N_{ch}$ = 6. The data obtained at the half-activation of the channels (i.e., drift = A = 0). Red—no cooperation ($\Delta_{drift}$ = 0), blue—negative cooperation ($\Delta_{drift}$ = 0.1 kT/rcu; open state of a channel favors the closure of its neighbors), grey—weak positive cooperation ($\Delta_{drift}$ = −0.05 kT/rcu; open state of a channel favors the opening of its neighbors), dark grey—strong positive cooperation ($\Delta_{drift}$ = −0.1 kT/rcu). Prob. means probability.

Examining the entropy values for a fixed number of channels, it is generally observed that the maximum corresponds to the absence of cooperation. This occurs because the switching probability *P*, introduced above, is reduced by drift in the most frequently occupied conduction states. For instance, under positive cooperation, the probability (per single channel) to leave the state with all *N* channels “on” decreases. Consequently, long-lived open dwell times become more frequent, interlacing with short closed dwell times, while intermediate-length dwell times—otherwise contributing significantly to the entropy—are suppressed. Long-lived states remain rare, but they still determine $P_{\mathrm{open}}$ (Figure 9). In contrast, negative cooperation leads to a reduction in the probability of short dwell times, as multi-channel opening activity is suppressed. In the extreme case, where cooperation statistically allows only one channel in the cluster to conduct, that channel behaves like a single channel with a relatively broad dwell-time distribution, without the time-scale reduction described above.

Comparing the results of Shannon entropy addressed to the dwell times to the results obtained before for the currents, we do not observe any clear saturation effects related to cooperation. The metric in the space of the dwell times does not allow for distinguishing cooperation from no cooperation within a cluster, as it does in the space of currents.

**Figure 10:**
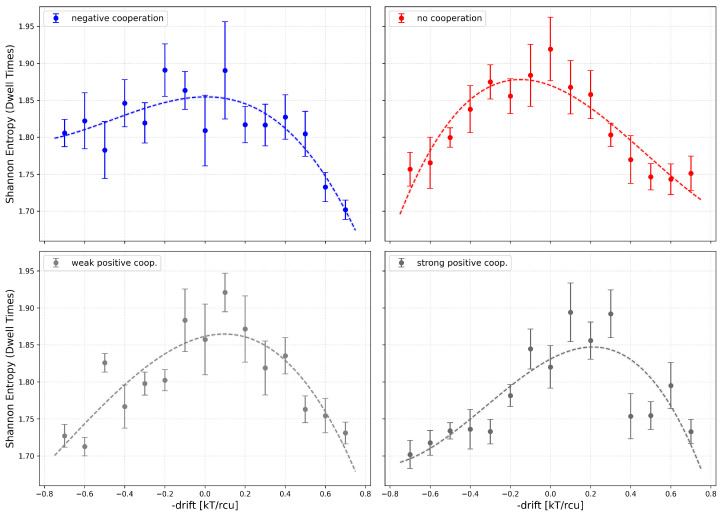
Shannon entropy of the dwell-time series of cluster states evaluated for a 4-channel cluster at different values of the global drift (representing the channel activation level by external stimuli, like, e.g., voltage, ligand concentration) and different strengths of the inter-channel cooperation. Red—no cooperation ($\Delta_{drift}$ = 0), blue—negative cooperation ($\Delta_{drift}$ = 0.1 kT/rcu; open state of a channel favors the closure of its neighbors), grey—weak positive cooperation ($\Delta_{drift}$ = −0.05 kT/rcu; open state of a channel favors the opening of its neighbors), dark grey—strong positive cooperation ($\Delta_{drift}$ = −0.1 kT/rcu). The errorbars depict the standard errors.

The effect of external channel activation on the Shannon entropy of the dwell times can be understood in the framework of the previous observations: we have a maximum of the entropy in the case of no net drift (including possible cooperation); outside this value, the entropy drops down (Figure 10).

### 3.3. Sample Entropy

#### 3.3.1. Effects of Window Length and Cluster Size on the SampEn Values

**Figure 11:**
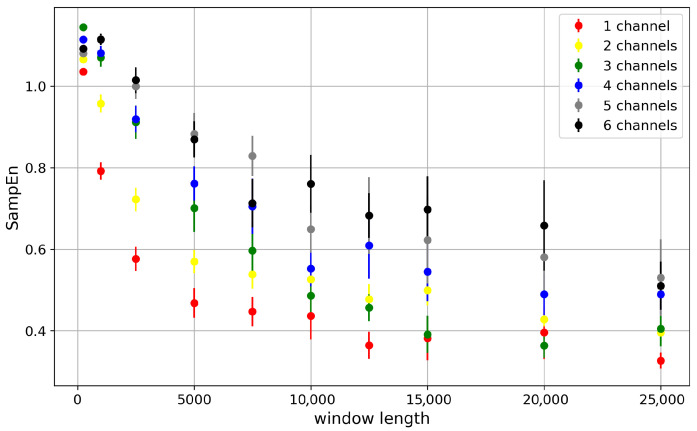
The dependency of average Sample Entropy (SampEn) describing dwell-time series of channel cluster states on window size at the sequence length *m* = 2. The presented *SampEn*s were evaluated for different numbers of channels in the cluster (depicted in different colors). No cooperation between the channels is assumed. The error is given by the standard deviation of a sample population (SE).

Since Sample Entropy requires the identification of sequences in the recording, and some statistical analysis of this data, it requires long recordings to develop the relevant probability distributions from the sample. The shape of the dependency in Figure 11 for all clusters reflects the gradual development of the exact dwell-time distribution with a longer window length. For short window lengths, the distribution is very coarse, with a high peak at short dwell times (well modeled by an exponential distribution in the Markovian approach) and zeros in the distribution tails. Such a structure of the dwell-time distribution overestimates the probabilities of short dwell times due to the lack of long dwell-time realizations. As they become recorded, the short dwell-time peaks reduce, giving rise to the distribution tails, and their entropic contributions grow.

Within each window length, entropy takes larger values when more channels occur in the cluster. That is because the dwell-time structure becomes richer if many channels are in action within the cluster. This happens even at half-activation, and even when the closed and open single-channel dwell times are probabilistically equal. Of course, the primary probability of leaving a conduction state is still roughly $N_{ch}\cdot P_{1}$ (where $P_{1}$ is the probability to switch the state of a single channel), but some corrections to this estimate are possible.

For example, if all *N* channels are “on”, the chance of leaving this state is related to the probability that at least one channel changes its state $N_{ch}\cdot P_{1}$. In contrast, considering a state with $N_{ch}/2$ channels “on”, there is a chance of ${\left(N_{ch}/2\right)}^{2}P_{1}^{2}$ that a change of the state of one of the channels in the cluster is accompanied by an opposite change of some other channel. The probability of leaving this conduction state is thus less than $N_{ch}\cdot P_{1}$, and so it is possible to find minor differences between dwell times generated by different conduction states, even at half-activation.

The compromise between the availability of experimental data in typical patch-clamp recordings and the reliability of SampEn estimation (obtained while reaching an abundant population of dwell-time sequences, which describes sufficiently well the variability and relative incidence of different types of dwell-time sequences) is that we have chosen 5000-point windows for further calculations.

#### 3.3.2. Effects of Inter-Channel Cooperation Strength and Mode on the SamEn Values

**Figure 12:**
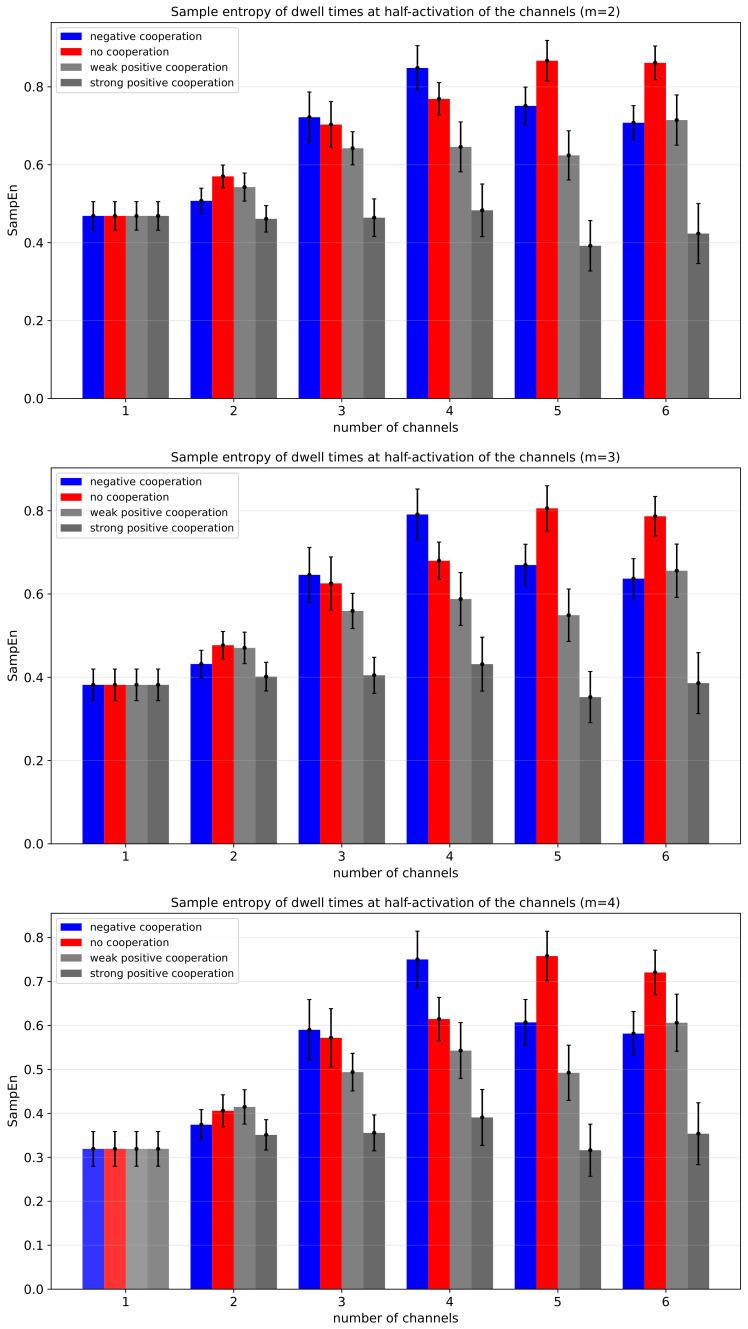
Sample Entropy of dwell times of cluster states evaluated for different cluster sizes and different strengths of the inter-channel cooperation. SampEn was calculated for the segment-length parameter *m* equal to 2 (**top panel**), 3 (**middle panel**), and 4 (**bottom panel**) at the half-activation of the channels (i.e., for drift = *A* = 0). Red—no cooperation ($\Delta_{drift}$ = 0), blue—negative cooperation ($\Delta_{drift}$ = 0.1 kT/rcu; open state of a channel favors the closure of its neighbors), gray—weak positive cooperation ($\Delta_{drift}$ = −0.05 kT/rcu; open state of a channel favors the opening of its neighbors), dark gray—strong positive cooperation ($\Delta_{drift}$ = −0.1 kT/rcu). The green hatched bar indicates the results for one channel.

Figure 12 shows the Sample Entropy (SampEn) of dwell times at half-activation, calculated for three different segment length parameters *m* = 2, 3, 4, as a function of the number of channels for different modes and strengths of inter-channel cooperativity. In the case of independent channel gating (no cooperation), an increase in the cluster size leads to a pronounced gain in SampEn. It reflects higher local temporal complexity of the dwell-time series of the multi-channel cluster states due to random switching “on” and “off” of the $N_{ch}$ channels. The more non-cooperating channels in the cluster, the less predictable the signal on a short time-scale.

**Figure 13:**
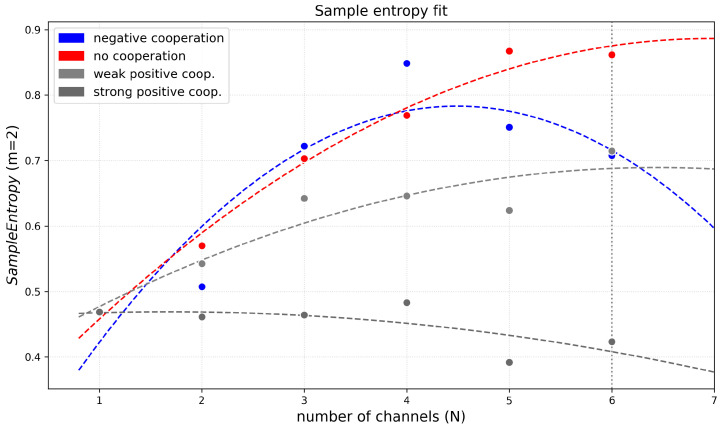
The parabolic fittings of the recognized dependencies of Sample Entropy of cluster currents vs. the number of co-assembled channels for different modes of cooperation (at *m* = 2). The data obtained at the half-activation of the channels (i.e., drift = A = 0). Red—no cooperation ($\Delta_{drift}$ = 0), blue—negative cooperation ($\Delta_{drift}$ = 0.1 kT/rcu; open state of a channel favors the closure of its neighbors), grey—weak positive cooperation ($\Delta_{drift}$ = −0.05 kT/rcu; open state of a channel favors the opening of its neighbors), dark grey—strong positive cooperation ($\Delta_{drift}$ = −0.1 kT/rcu). The coefficients of fit are presented in Appendix A.

Introduction of the inter-channel cooperativity modifies the $SampEn(N_{ch})$ function (Figure 12 and Figure 13, Table A3). For the negative cooperativity, SampEn increases with the number of channels and reaches a maximum for four-channel clusters, and then, for a larger number of channels, SampEn decreases. The initial rise in Sample Entropy with $N_{ch}$ reflects the higher local temporal complexity of cluster states due to weak opening-counteracting effects that even entangle the purely random behavior. When cluster size is sufficiently big ($N_{ch}$ = 5 or $N_{ch}$ = 6), the additive effects of inter-channel negative cooperation result in some synchronization, which reduces local unpredictability, causing Sample Entropy to level off or even decrease. In other words, initial growth in the number of channels delivers more conduction states with individual dwell-time characteristics, enlarging the entropy. At a threshold value of $\hat{N}$, there develops a condition for the maximum number of activated channels within a cluster (where larger activation is prevented by the negative cooperation). Escape from this conduction state is reduced by $\hat{N}$ compared to the single-channel conduction state, no matter the actual $N>\hat{N}$. In contrast, the activation from a non-conducting state is proportional to the actual number of channels in a cluster, *N*. This brings asymmetry to the dwell-time distribution (long $∼1/\hat{N}$ vs. short ∼1/N), and given the similarity threshold of 20% of the standard deviation, the distribution of short dwell times becomes indistinguishable, leading to the entropy decrease. A similar trend can be seen for strong cooperation, and may be explained by the same arguments by assuming a minimum number of activated channels in a cluster $\hat{N}$.

Generally speaking, the weak and strong positive cooperativity result in systematically lower SampEn values across all channel numbers in comparison to the non-cooperating clusters. Increasing the segment (pattern) length within the *m* parameter range from 2 to 4 does not influence these qualitative observations (thus, for short signal patterns, adding another state to a sequence, generally, neither distorts the existing correlations nor strengthens the recognized trends). Naturally, strong positive cooperativity shows the weakest dependence on the number of channels. That is because the concerted $N_{ch}$-dependent opening-reinforcing effects mitigate the effects of global cluster state intersection by random individual ’on’ and ’off’ switching. The strength of positive cooperation between channels within the cluster easily translates to a reduction in Shannon Entropy compared to the no cooperation mode, due to the cooperation–strength-dependent propensity of the system to become more globally ordered, leading to a situation where certain state combinations dominate. Overall, the SampEn results (Figure 12 and Figure 13, Table A3) indicate that SampEn describing dwell-times of cluster states (regardless of the *m* parameter in the range 2–4) may be used for a clear separation between different cluster cooperation modes and strengths.

**Figure 14:**
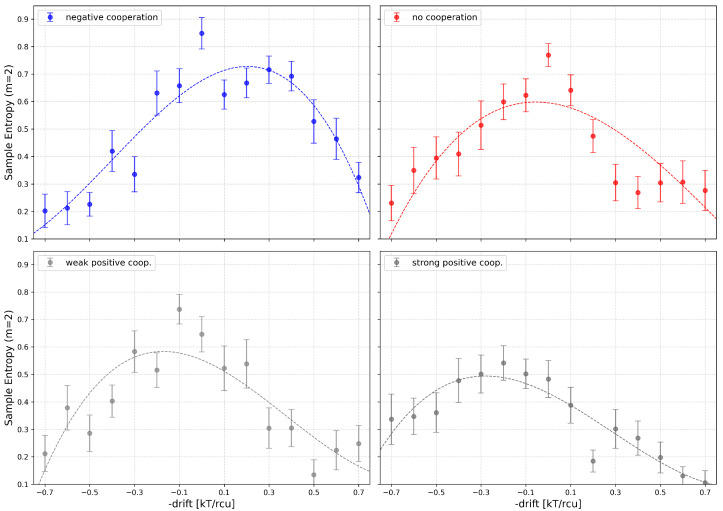
The dependency of SampEn on the drift force for 4-channel cluster cooperating with different values of the global drift (representing the channel activation level by external stimuli, like, e.g., voltage, ligand concentration) and different strengths of the inter-channel cooperation. Red—no cooperation ($\Delta_{drift}$ = 0), blue—negative cooperation ($\Delta_{drift}$ = 0.1 kT/rcu; open state of a channel favors the closure of its neighbors), grey—weak positive cooperation ($\Delta_{drift}$ = −0.05 kT/rcu; open state of a channel favors the opening of its neighbors), dark grey—strong positive cooperation ($\Delta_{drift}$ = −0.1 kT/rcu). Each plot contains the results calculated for *m* = 2. The errorbars depict the standard errors of the mean.

Considering the changes in SampEn as a result of global cluster activation, one can observe an expected trend to shift the maxima of Sample Entropy vs. drift to the right at negative cooperation and to the left at positive cooperation (Figure 14).

Except from shifting of the maxima one can also observe another effect: lifting of the curve on the diagram in the side, where drift opposes cooperation effects. This occurs because cooperation brings high diversity to the dwell times of different conduction states, which manifests in elevated entropy when the drift allows for different scenarios than “fully inactivated” or “fully activated” clusters. One should keep in mind here that activation bias (the activation drift) cannot compensate for cooperation drift on all conduction states at once. The range of choices for the compensating drift is reflected in broader elevated entropy values over this drift range.

## 4. Discussion

Studies on the cooperative gating phenomenon represent one of the most promising and relatively novel directions in channel-oriented research. This growing scientific interest arises from the phenomenon’s considerable significance in elucidating the molecular mechanisms that shape the multi-channel ionic current fluxes and the impact of collective effects on interesting features of channel-mediated electrochemical signaling, such as gating modalities, short- and long-term memory effects, and hysteresis in cluster currents at different levels of global cluster activation by external factors (like hysteresis in IV-curve of voltage-gated channel clusters) [24,25,58,76]. A deeper understanding of these processes is also essential for refining fundamental biophysical theories, including the extension of the core concepts, such as the qualitative description of action potential generation, which may fundamentally alter our understanding of neuronal encoding. For example, incorporating sodium channel cooperation into the Hodgkin–Huxley model improves the accuracy of descriptions of neuronal behavior [77,78]. In fact, by understanding the role of cooperative gating in neurons, we may discover new and important paradigms for the models of artificial neural networks to further improve AI technology. Continued progress in this area is expected to drive advances in channel-targeted pharmacology, particularly in developing approaches to modulate channel clustering and inter-channel cooperation, as proposed in recent studies [79].

Studies involving multi-channel recordings require dedicated and well-tailored methodologies for data analysis. Such approaches should either be newly developed or carefully adapted from existing analytical frameworks with appropriate validation. In this work, we showed how the entropic analysis can effectively capture the complex characteristics of multi-channel data. In particular, we demonstrated the applicability of two information entropy measures, Shannon entropy and Sample Entropy, to the analysis of two types of multi-channel data, which are easily obtainable experimentally. These include (i) idealized cluster current series, where each current amplitude corresponds to the number of simultaneously open channels [73,74,75], and (ii) the associated dwell-time series representing the duration of specific, consecutive cluster conduction states. These datasets were generated in this work through simulations based on a previously introduced simple random walk model [70], extended in this work to describe a multi-channel system. This approach allowed us to minimize the noise inherent in experimental data and to simplify the underlying gating mechanisms. It also enables straightforward interpretation of the resulting entropic characteristics of multi-channel systems (including concepts like dwell-time variability in half-activation due to switching asymmetries, which otherwise would probably be attributed to noise). Moreover, because the model relies on straightforward assumptions, it facilitates the formulation of general insights that can be compared with experimental observations across various cooperative channel systems—formed by channels of different types and governed by diverse molecular gating mechanisms. According to our results, the occurrence of cooperation in gating modifies the dependency of dwell times’ SampEn or currents’ Shannon entropy on the cluster size and the strength of channel-activating external factors (represented by the drift in the model). We are convinced that these general trends can also be present in the empirical patch-clamp recordings. Thus, our results can serve as benchmarks for probing real collective gating in ion channel clusters or simple biomimetic multi-nanopore systems, where some channel-specific deviations from the idealized system characteristics are expected. These deviations can, in turn, enable a more detailed characterization of individual channel systems and focus on channel-specific signal features.

So far, entropic analysis has been successfully employed to describe the gating of the ion channel both at the single-channel level [62,65,68] and at the whole-cell level [67]. In the present study, Shannon entropy and Sample Entropy are applied for the first time to multi-channel data, where multiple channels form a cooperative cluster. Our findings indicate that changes in entropy with cluster size or the strength of external channel-activating factors provide a powerful quantitative criterion for classifying data according to the mode and strength of inter-channel cooperativity. Specifically, the relationship between the Shannon entropy of total idealized currents and the increasing number of co-assembled channels ($N_{ch}$) under fixed external conditions strongly depends on the underlying cooperation mode. A monotonic increase in Shannon entropy with $N_{ch}$ suggests the absence of cooperativity, whereas an initial increase followed by stabilization of entropy at larger $N_{ch}$ values indicates the presence of cooperative interactions among channels. Moreover, the earlier this stabilization occurs, the stronger the inferred inter-channel cooperation. In the above, Shannon entropy proves considerably less effective in distinguishing cooperative dynamics when applied to dwell-time series of cluster states. This limitation arises because with the increase of channels in a cluster, the effective number of conduction states (affecting the entropy of the current) stabilizes, while the dwell times continue to be modulated by new channel units (like N+1 channels can perturb a conduction state faster than *N* channels).

The dwell-time series of a single ion channel represents a sequence of transitions between conformational states of varying stability, with only a limited number of possible target conformations originating from some initial conformation. Consequently, such data tend to exhibit distinct internal patterns that primarily arise from recurrent transition schemes between preferred conformational states. When additional channels are introduced, these characteristic patterns become disrupted due to the stochastic and intermittent opening and closing of neighboring channels, which introduce a degree of decorrelation into the system. However, this loss of correlation can be partially mitigated or even eliminated when collective gating behavior induces synchronization among channel states. This reasoning motivated the application of an alternative information-theoretic measure, Sample Entropy, which is particularly sensitive to the presence of repetitive sequences within time series data. Accordingly, we employed Sample Entropy to analyze the dwell-time series of channel cluster states, aiming to better capture the underlying temporal regularities associated with cooperative gating phenomena [59,60,62].

Analogously to the Shannon entropy of the total idealized current series, the shape of the dependency describing the changes in Sample Entropy of dwell times with the cluster size or the strength of external channel-activating factors turned out to be very sensitive to the existence, modality, and intensity of inter-channel cooperation. The observed dependence of Sample Entropy on the number of channels forming a cluster reflecting the mode and strength of the inter-channel cooperation manifests the interplay between interaction-induced variability and collective ordering in the system.

As shown for a negatively cooperating four-channel cluster at its half-activation by external factors (Figure 12 and Figure 13), the initial increase in SampEn indicates that adding channels introduces stochastic disruption of the single-channel dwell times’ correlations that enhance local temporal complexity. The subsequent saturation and decrease in SampEn for larger numbers of channels suggest the emergence of collective dynamics and partial synchronization, which reduce local unpredictability by constraining the accessible temporal patterns. As explained before, this happens mostly due to the separation of the dwell-time scales between fast and slow events. For negatively cooperative systems, this implies that temporal complexity reaches its maximum at intermediate cluster sizes, beyond which collective ordering becomes dominant and suppresses variability. In contrast, strong positive cooperativity promotes synchronization even at relatively small channel numbers, leading to reduced temporal complexity and lower SampEn values compared with non-cooperative clusters. In such cases, the strong coupling among channels suppresses irregular temporal behavior, resulting in a weakened dependence of SampEn on cluster size. Weak positive cooperativity, on the other hand, introduces a modest degree of ordering that slightly reduces SampEn relative to the non-cooperative case, reflecting mild temporal structuring without full synchronization.

When relating the present results to real multi-channel systems, we anticipate that the main trends in Shannon and Sample Entropy observed in our simulations, as well as the mechanisms proposed to drive them, will have clear counterparts in experimental data. Specifically, when comparing the activity of cooperative versus non-cooperative channel clusters, such as those formed by different channel mutants under identical external conditions and with the same number of functionally active channels within a patch, distinct entropy values are expected. Furthermore, by analyzing how entropy varies with cluster size or with the strength of channel-activating stimuli, one can infer the interplay between the signal components that contribute to temporal ordering (e.g., synchronization induced by cooperativity or external activation) and those responsible for stochastic randomization (e.g., independent opening and closing of channels within a cluster). We may also draw conclusions about the strength of cooperative interactions by examining the entropy maximum as a function of activating stimulus strength, as well as the potential nonlinear dependence of cooperation on both the stimulus and the number of channels. Together with classical measures, such as NPo with statistically sound estimates of the number of channels $N_{ch}$, such relationships can significantly facilitate the rational modeling of collective gating phenomena and guide the interpretation of empirical patch-clamp data. In the long term, these insights may not only deepen our understanding of nanoscale transport phenomena in biological multi-channel systems [24] or correlate to some physiological and pathological phenomena, but can also be helpful in the design of advanced multi-nanopore biomimetic nanofluidic devices [80] or novel activation functions for neurons in artificial neural network technologies [78]. The presented analytical framework can also be extended beyond cooperativity among channels of the same type to other forms of cooperative behavior. These include cooperativity between subunits within a single channel, such as during the allosteric activation of BK channels [81] and in other channels [48,49,50], as well as the concerted action of ion channels of different types [51,52]. Such interactions can also be examined using patch-clamp current recordings as input data, where the symptoms of collective behavior may be reflected in dwell-time distributions.

## 5. Conclusions

The Shannon entropy of total idealized currents and the Sample Entropy of dwell-time series in multi-channel clusters effectively capture the transition from independent multi-channel behavior to cooperation-induced temporal ordering. These entropy-based measures can therefore serve as efficient diagnostic indicators of inter-channel cooperativity within clustered ion channel systems and complement classical descriptions of multi-channel systems based on kinetic parameters. The results presented here, derived from a simplified model, provide a useful reference framework that can be directly compared with empirical observations from cooperative channel assemblies.

## Data Availability

The research data are available at https://doi.org/10.5281/zenodo.18042547, accessed on 24 December 2025.

## Abbreviations

The following abbreviations are used in this manuscript:

$D_{RC}/D_{B}$	Ratio between the diffusion coefficients of the reaction coordinate and the boundaries
$N_{ch}$	Number of co-assembled channels forming a cluster
RC	Reaction coordinate
SampEn	Sample entropy
TP	Threshold separating open and closed channel states
*U*	Potential function

## Appendix A. Fitting Parameters of the Recognized Dependencies of Shannon and Sample Entropy on the Number of Channels in the Cluster

**Table A1:** The parameters of parabolic fit of the Shannon entropy of cluster currents (ShEn) vs. cluster size ($N_{ch}$) of form $ShEn=a\cdot N_{ch}^{2}+b\cdot N_{ch}+c$ together with the corresponding inaccuracies of the *a*, *b* and *c* coefficients (Δa, Δb, Δc) at different modes and strengths of the inter-channel cooperation. The value of peak $N_{ch}$ describes the $N_{ch}$ for which the maximal ShEn is reached. The input data obtained at the half-activation of the channels (i.e., drift = A = 0). Red—no cooperation ($\Delta_{drift}$ = 0), blue—negative cooperation ($\Delta_{drift}$ = 0.1 kT/rcu; open state of a channel favors the closure of its neighbors), grey—weak positive cooperation ($\Delta_{drift}$ = −0.05 kT/rcu; open state of a channel favors the opening of its neighbors), dark grey—strong positive cooperation ($\Delta_{drift}$ = −0.1 kT/rcu).

	*a*	Δa	*b*	Δb	*c*	Δc	Peak $N_{\mathrm{ch}}$
no cooperation	−0.0423	0.0022	0.5527	0.0158	0.5181	0.0242	6.53
weak positive coop.	−0.0587	0.0187	0.5974	0.1335	0.4955	0.2040	5.09
strong positive coop.	−0.0373	0.0240	0.3214	0.1713	0.7733	0.2618	4.31
negative coop.	−0.0322	0.0095	0.4132	0.0677	0.6223	0.1035	6.42

**Table A2:** The parameters of the fitting of the Shannon entropy of dwell times ($ShEn_{dw}$) vs. cluster size ($N_{ch}$) by the function of form $ShEn_{dw}=a/N_{ch}+b$ together with the corresponding inaccuracies of the *a* and *b* coefficients (Δa, Δb) at different modes and strengths of the inter-channel cooperation. The input data obtained at the half-activation of the channels (i.e., drift = A = 0). Red—no cooperation ($\Delta_{drift}$ = 0), blue—negative cooperation ($\Delta_{drift}$ = 0.1 kT/rcu; open state of a channel favors the closure of its neighbors), grey—weak positive cooperation ($\Delta_{drift}$ = −0.05 kT/rcu; open state of a channel favors the opening of its neighbors), dark grey—strong positive cooperation ($\Delta_{drift}$ = −0.1 kT/rcu).

	*a*	Δa	*b*	Δb
no cooperation	0.6564	0.0763	1.7317	0.03804
weak positive coop.	0.7471	0.0665	1.6638	0.0332
strong positive coop.	0.8032	0.0630	1.6120	0.0314
negative coop.	0.6877	0.0775	1.7130	0.0386

**Table A3:** The parameters of parabolic fit of the Sample Entropy of dwell times (SampEn) vs. cluster size ($N_{ch}$) of form $SampEn=a\cdot N_{ch}^{2}+b\cdot N_{ch}+c$ together with the corresponding inaccuracies of the *a*, *b* and *c* coefficients (Δa, Δb, Δc) at different modes and strengths of the inter-channel cooperation. The value of peak $N_{ch}$ describes the $N_{ch}$ for which the maximal SampEn is reached. The input data obtained at the half-activation of the channels (i.e., drift = A = 0). Red—no cooperation ($\Delta_{drift}$ = 0), blue—negative cooperation ($\Delta_{drift}$ = 0.1 kT/rcu; open state of a channel favors the closure of its neighbors), grey—weak positive cooperation ($\Delta_{drift}$ = −0.05 kT/rcu; open state of a channel favors the opening of its neighbors), dark grey—strong positive cooperation ($\Delta_{drift}$ = −0.1 kT/rcu).

	*a*	Δa	*b*	Δb	*c*	Δc	Peak $N_{\mathrm{ch}}$
no cooperation	−0.0120	0.0038	0.1678	0.0269	0.3021	0.0411	6.97
weak positive coop.	−0.0072	0.0065	0.0926	0.0468	0.3915	0.0715	6.44
strong positive coop.	−0.0033	0.0052	0.0109	0.0369	0.4600	0.0563	1.67
negative coop.	−0.0296	0.0122	0.2659	0.0870	0.1859	0.1329	4.49
